# Isophorone Diisocyanate (IPDI) Microencapsulation for Mono-Component Adhesives: Effect of the Active H and NCO Sources

**DOI:** 10.3390/polym10080825

**Published:** 2018-07-26

**Authors:** Mahboobeh Attaei, Mónica V. Loureiro, Mário do Vale, José A. D. Condeço, Isabel Pinho, João C. Bordado, Ana C. Marques

**Affiliations:** 1 CERENA, Departamento de Engenharia Química, Instituto Superior Técnico, Universidade de Lisboa, Avenida Rovisco Pais, 1049-001 Lisboa, Portugal; mahboobehattaei@tecnico.ulisboa.pt (M.A.); monica.loureiro@tecnico.ulisboa.pt (M.V.L.); mario.vale@tecnico.ulisboa.pt (M.d.V.); jose.condeco@tecnico.ulisboa.pt (J.A.D.C.); jcbordado@tecnico.ulisboa.pt (J.C.B.); 2 CIPADE—Indústria e Investigação de Produtos Adesivos, SA. Av. 1° de Maio, 518 Z. Industrial 1, Apt. 82, 3701-909 São João da Madeira, Portugal; adhesives@cipade.com

**Keywords:** microcapsules, isocyanate, IPDI, 3-isocyanatopropyltriethoxysilane, microemulsion, interfacial polymerization, controlled release, adhesives, mono-component, eco-innovative, footwear

## Abstract

Polyurea/polyurethane (PUa/PU) shell microcapsules (MCs), containing high loadings of isophorone diisocyanate (IPDI) in the core, were developed to enable the production of mono-component, eco-friendly and safer adhesive formulations for the footwear industry. IPDI microencapsulation was obtained via oil–in–water (O/W) microemulsion combined with interfacial polymerization. A methylene diphenyl diisocyanate (MDI) compound (a commercial blend of monomeric and polymeric species), with higher reactivity than IPDI and low viscosity, was added to the O phase to competitively contribute to the shell formation, improving its quality. Four different active H sources were tested, aimed at achieving a high encapsulation yield. The successful encapsulation of IPDI was confirmed by Fourier transformed infrared spectroscopy (FTIR) and thermogravimetric analysis (TGA), while the MCs’ morphology and size distribution were assessed by scanning electron microscopy (SEM). The incorporation of a multifunctional isocyanate silane in the O phase, as “latent” active H source, led to the formation of impermeable PUa/PU-silica hybrid shell MCs with more than 60 wt.% of pure encapsulated IPDI. A proof-of-concept study shows high peeling strength and a structural type of failure of the adhesive joint, revealing an effective IPDI release. These new engineered MCs are found to be promising crosslinkers for mono-component adhesives for high demanding applications.

## 1. Introduction

Isocyanates have been used for over 60 years in polyurethane (PU) and polychloroprene adhesives formulations, mainly because of their high reactivity and capability to promote a good adhesion. The formation of PUs occurs by the reaction between the extremely reactive polyisocyanates’ NCO groups and any chemical group that contains an active hydrogen (H) atom [1]. Current commercial polyurethane-based adhesives are bicomponent, i.e., they are composed by two components, a polyol base (OH prepolymer) and a crosslinker. High quality, strong and long-lasting adhesives used in the footwear industries are typically isocyanate-based, which provides appropriate high strength quality adhesive joints when gluing the different parts of the footwear. However, the high toxicity of isocyanate compounds is restricting their use in the industry, based on current legislation. Companies in the adhesive sector are therefore willing to offer a safe adhesive solution to these industries but still based on isocyanate chemistry due to its high adhesive efficiency. This work refers to the synthesis of microcapsules (MCs) with a polyurea/polyurethane (PUa/PU) shell containing isophorone diisocyanate (IPDI), as the encapsulated isocyanate, to be added to footwear adhesives formulations. The encapsulation of isocyanate, the crosslinker, enables the production of a new generation of one component adhesives, contributing to an advance of the state of the art in what regards adhesive technology and an improvement of the productivity in the footwear industry. These novel one-component adhesives will contain encapsulated isocyanates which decreases the risk to health by avoiding the user’s contact with this toxic chemical. In addition, these adhesives will not require the weighing and mixing of components, which prevents manufacturing errors and reduces packaging by 50%, decreasing its ecological footprint.

In this work, the IPDI was encapsulated using an interfacial polymerization method in combination with an oil–in–water (O/W) microemulsion technique. Microencapsulation can be defined as a way of coating small particles, liquid droplets or gas bubbles within a thin film or shell, which protects or isolates the core material from the surrounding environment. Many different active materials such as drugs, enzymes, vitamins, pesticides, flavors, and catalysts have been successfully encapsulated inside shells made from a variety of polymeric and non-polymeric materials, depending on the end use of the encapsulated products. 

**Figure 1:**
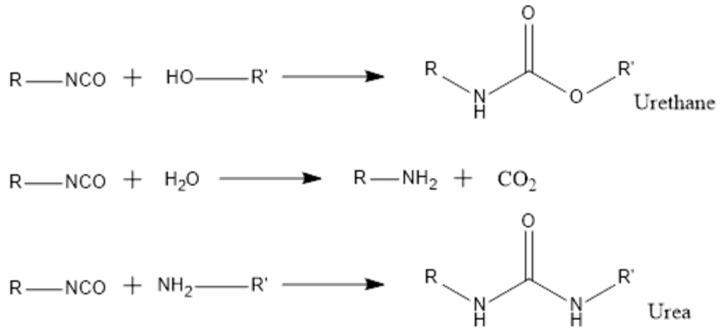
The reaction of isocyanate with hydroxyl group, water, and amino group.

The employed technique involved an emulsification process in combination with interfacial polymerization. The emulsion is obtained through vigorous stirring of two or more immiscible phases composed by at least two liquids, usually in the presence of an emulsifier. Regarding the interfacial polymerization process, it is composed by two steps. During the first step, the diffusion of two different reactive monomers occurs, and they come in contact at the interface, i.e., at the surface of the oil droplets of the emulsion system, leading to the formation of an initial thin polymeric layer shell [2]. During the second step, an increase of the shell thickness occurs, by the continuation of the polymerization reactions towards the organic (dispersed) phase. The polymerization is controlled by diffusion, so the growth’s speed of the MCs’ shell will decrease as the shell thickness increases [3]. The shell formation is due to the reaction between the –NCO groups from isocyanate and active H sources present in the aqueous phase of the emulsion system, which may be provided by amines (NH groups) or polyols (OH groups). Different active H sources may have different chain length and chemical structure and, therefore, different steric hindrance and reactivity, which strongly influences the MCs’ shell thickness, as well as their morphology [4]. As represented in Figure 1, the reaction between –NCO groups and different active H sources can lead to different final compounds: when reacting with –OH groups, it leads to the formation of a urethane linkage; with water it leads to the formation of an amine; and with amino groups it leads to the formation of a urea linkage.

The major efforts already reported for microencapsulation of isocyanate compounds belong to the encapsulation of monomeric isocyanate IPDI, as a healing agent. Yang et al. reported the successful microencapsulation of IPDI, as a healing agent, via interfacial polymerization, with a microencapsulation yield of 70 wt.%, taking into account the amount of IPDI and solvent [5]. In their work, a prepolymer was previously prepared using a solution of toluene diisocyanate (TDI) in cyclohexanone, followed by further addition of 1,4-butanediol as an active H source. Carefully analyzing the paper, the encapsulated isocyanate yields at ca. 35 wt.% of the MCs’ weight. The encapsulation of IPDI within MCs’ shells composed of poly(urea-formaldehyde), polyurethane, polyurea, bi-layer polyurethane/poly (urea-formaldehyde) and a double-layered shell composed of polyurea/melamine formaldehyde has already been reported [6,7,8]. Different compounds have also been tested as active H sources, for example poly vinyl alcohol, glycerol, 1,4-butanediol and 1,6-hexanediol [9,10,11]. Some of these authors reported on the impossibility of achieving MCs using commercial pre-polymers [8] and employed pre-polymers which had to be synthesized on purpose, for promoting the shell formation. In addition, other authors have reported on shelf-life issues, solvent resistance issues, or weak mechanical performance of the shells. Moreover, the yield of isocyanate encapsulation is relatively poor in most of the cases found in the literature. Indeed, the encapsulation efficiency is sometimes referred to the encapsulated isocyanate and solvent, instead of the pure isocyanate only.

In the present work, IPDI was efficiently microencapsulated using a readily commercially available MDI source, Ongronat^®^ 2500, which consists of a mixture of monomeric and polymeric MDI species, responsible for an acceptable low viscosity of the dispersed phase of the emulsion and, therefore, no solvent was required. It displays significantly higher reactivity than IPDI, favorably competes in the formation of the polymeric shell, enhancing the encapsulated IPDI content, and has not yet been described in the literature for this application. In addition, four different active H sources were tested, namely 3-(2-aminoethylamino) propyltrimethoxysilane (aminosilane) in combination with tetraethyl orthosilicate (TEOS), diethylenetriamine (DETA) and 3-(triethoxysilyl) propyl isocyanate or 3-isocyanatopropyltriethoxysilane (IPES), to achieve a faster polymerization reaction with an increased encapsulation yield, as well as durable (long shelf-life) MCs. The novelty of this work relies on: (i) optimization studies involving multifunctional active H sources and a commercially available high reactivity and low viscosity isocyanate compound (Ongronat^®^ 2500), which eliminates the need for a solvent and increases the encapsulated pure isocyanate content; (ii) the use of aminosilane and TEOS in the synthesis, which not only contributed as an active H source, but also for a PUa/PU-silica hybrid shell; (iii) the addition of a “latent” active H source, isocyanato silane (IPES), which has both a Si-ethoxy (Si–OC_2_H_5_) and isocyanato functionality and, which can be placed in the dispersed, organic phase, contrary to common active H sources; and (iv) the application of MCs obtained through interfacial polymerization, containing IPDI, as a crosslinking agent to be applied in the adhesive industry for high demanding applications.

The work reported in this paper was carried out within the framework of a Technology Platform on Microencapsulation and Immobilization led by A.C. Marques, which deals with the synthesis of MCs, microscaffolds and microspheres with tailored morphologies, organic functionalities and a wide porosity range [12,13,14,15,16].

## 2. Materials and Methods

### 2.1. Materials and Methods

#### 2.1.1. Materials

The MDI compound and shell precursor Ongronat^®^ 2500 was purchased from BorsodChem. The isocyanate to be encapsulated, Isophorone diisocyanate (IPDI, with 98% purity), was obtained from Bayer. Regarding the active H source compounds, the 3-(2-aminoethylamino) propyltrimethoxysilane (with 90% purity) and tetraethyl orthosilicate (TEOS, with 99% purity) were purchased from VWR Chemicals; Diethylenetriamine (DETA) (with 99% purity) was obtained from Alfa Aesar; and the 3-isocyanatopropyltriethoxysilane (IPES, with 95% purity) was purchased from Aldrich Chemistry. The surfactant Gum Arabic (GA) was obtained from Labchem. All chemicals were used as received, without further purification.

#### 2.1.2. Preparation of Microcapsules

The oil–in–water (O/W) emulsion system was obtained through vigorous stirring of the isocyanates, as the oil phase, consisting of 6.7 wt.% of the total emulsion system, with the water phase consisting of 95.3 wt.% Milli-Q water and 4.7 wt.% GA, at a stirring speed of 3200 rpm with an Ultra-Turrax (IKA T25 digital ULTRA TURRAX, Germany) during 10 min at room temperature (RT). The oil phase is composed by two isocyanates, being IPDI, present at 70 wt.%, the one to be encapsulated and Ongronat^®^ 2500, present at 30 wt.%, the one to competitively contribute to the MCs’ shell formation. The IPDI is less reactive than Ongronat^®^ 2500, which allows its encapsulation due to the faster reaction of Ongronat^®^ 2500 with the OH groups of the aqueous phase.

Once the emulsion was ready, the active H sources (aminosilane, TEOS and DETA) were added, dropwise, to the emulsion system (W phase) while under mechanical stirring at 400 rpm, using a mechanical stirrer Heidolph RZR 2051 control. IPES, on the other hand, was added to the O phase used to prepare the emulsion. The emulsification and stirring rates had been previously optimized for these microencapsulation syntheses. During this procedure, the temperature was increased gradually from RT to the maximum synthesis temperature, listed in Table 1. The reactional medium was maintained at the maximum temperature, under mechanical stirring for several hours, depending on the synthesis, until the MCs shell attains enough maturity to tolerate the pressure applied during the filtration procedure. The MCs were then filtrated using a vacuum filtration system, while washed with Milli-Q water. The final product was dried at atmospheric pressure and room temperature for 24 h.

It should be noted that these syntheses require a very frequent monitoring of the emulsion quality by means of optical microscopy, in order to control the stability of the emulsion, as well as the maturity degree of the shell.

### 2.2. Characterization

The adopted techniques and methods for the MCs’ characterization include optical microscopy, scanning electron microscopy (SEM), Fourier transformed infrared spectroscopy (FTIR) and thermogravimetric analysis (TGA). The photomicrographs obtained from SEM were also used to evaluate the MCs’ size distribution using the software ImageJ. Proof of concept studies were carried out to evaluate the MCs’ performance as encapsulating agents to enable mono-component and self-reactive adhesives.

#### 2.2.1. Optical Microscopy

A Kruss, MSZ 5600 optical microscope was used to evaluate the MC’ size, shape and maturity (stiffness, qualitatively assessed by punching, or tearing with the point of a needle), as well as the stability of the emulsion and droplets’ size during the synthesis procedure.

#### 2.2.2. Scanning Electron Microscope (SEM)

SEM analysis was carried out to evaluate the morphology, size distribution, roughness and porosity of the obtained MCs, using a JEOL JSM7001F (JEOL, Tokyo, Japan) with a FEG-SEM (Field Emission Gun) system. Previously to the analysis, the MCs were placed in a sample holder using conductive adhesive tape with a double face. Afterwards, the samples were coated with a conductive Au/Pd thin film, through sputtering, using a Quorum Technologies sputter coater model Q150T ES.

To evaluate the MCs’ size distribution, the images obtained through SEM were imported to the software ImageJ (v 1.51p, National Institutes of Health, Bethesda, MD, USA). After calibrating the scale, the contrast of the SEM images was enhanced, and the MCs were highlighted by a thresholding function. After treating the images to remove residual debris and agglomerates, the data for the size distribution of MCs was obtained using the Analyze Particles functionality of the software.

#### 2.2.3. Fourier Transformed Infrared Spectroscopy (FTIR)

FTIR characterization was used to study the chemical structure of the obtained MCs by identification of specific characteristic groups, in order to assess the presence of certain compounds in the MCs’ shell and core. This technique enabled to confirm the presence of unreacted NCO groups in the MCs’ core, as well as to make a comparative evaluation of the encapsulated NCO content among different syntheses. A PerkinElmer, Spectrum Two, FTIR spectrometer was used, equipped with a Pike Technologies MIRacle^®^ Attenuated Total Reflectance (ATR) accessory. The spectra were achieved with a resolution of 4 cm^−1^ and data collection of 8 scans.

#### 2.2.4. Thermogravimetric Analysis (TGA)

Through TGA analysis, it was possible to quantify the amount of encapsulated compound in the obtained MCs. The analyses were performed under a controlled nitrogen atmosphere, at a temperature increase rate of 10 °C·min^−1^ using a TGA HITACHI STA 7200.

#### 2.2.5. Peeling Strength Test

The peeling strength test was essential to evaluate the adhesive effectiveness of the adhesive joint, in the bonding process. Peeling strength is the average load per unit width of the bond, required to separate bonded materials. A separation angle of 180 degrees was applied. A high peeling strength value, i.e., similar to that of the reference sample (adhesive joint with non-encapsulated IPDI), confirms the release of the microencapsulated IPDI content and its effective reaction with the surrounding environment (OH pre-polymer).

For this test, MCs with encapsulated IPDI (I MCs) were added to an adhesive formulation base component, OH pre-polymer (having OH groups available to react), supplied by CIPADE S.A., to act as a crosslinker. The quantity of MCs added to the solution was such that the quantity of the encapsulated isocyanate corresponds to 5 wt.% of the OH pre-polymer (base component). The resulting mono-component adhesive material was applied as a homogeneous thin film (0.2 mm of thickness) onto previously carded leather substrates. The film was allowed to dry at RT for 15 min. The substrates were then placed on top of each other, with the adhesive faces in contact and were subjected to the conditions of pressure and temperature used in the footwear industry in the adhesive application process. For this test, infrared radiation and a manual press with electrically heated platens, Carver, were used. The samples were left to stand for 48 h at RT before carrying out the peeling strength test. A universal testing machine Instron 5566 was used at a constant rate of speed of 100 mm/min. Tests using a reference sample, namely the same OH pre-polymer but with non-encapsulated IPDI, were also performed for results comparison.

## 3. Results and Discussion

**Figure 2:**
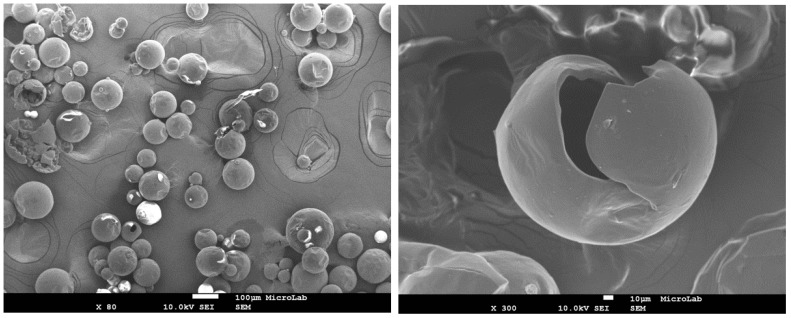
SEM image of AT MCs (Active H source: aminosilane and TEOS). The image on the right displays a broken MC which demonstrates the core–shell morphology typical of these MCs. Scale bar = 10 μm.

**Figure 3:**
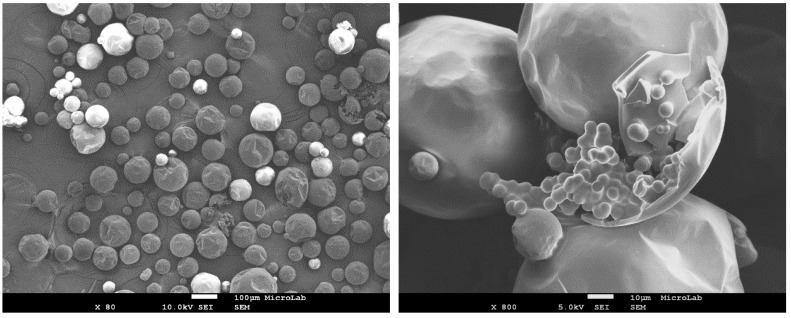
SEM image of D MCs (Active H source: DETA). The image on the right displays a broken MC which demonstrates the core–shell morphology typical of these MCs. Scale bar = 10 μm.

**Figure 4:**
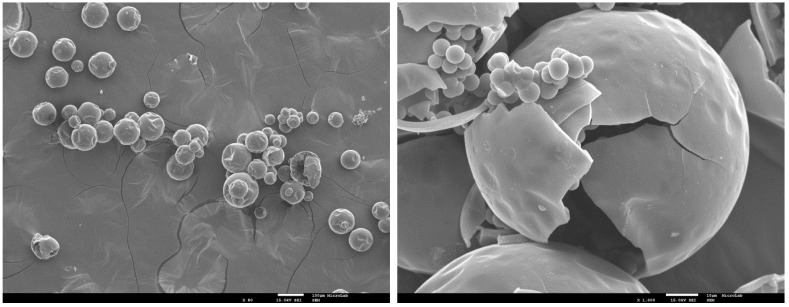
SEM image of I MCs (Active H and NCO source: isocyanato silane, IPES). The image on the right displays a broken MC which demonstrates the core–shell morphology typical of these MCs. Scale bar = 10 μm.

The morphology and size distribution of the MCs were assessed by SEM. Figure 2, Figure 3 and Figure 4 show the SEM photomicrographs of the AT MCs, D MCs and I MCs, respectively. Some of the MCs were crushed on purpose to make possible identifying their characteristic morphology, including their shell thickness. All the MCs present a core–shell morphology, have spherical shape and do not evidence significant aggregation. An average shell thickness of 8 ± 1, 2 ± 0.5 and 3 ± 0.5 μm was found for AT MCs, D MCs and I MCs, respectively. For all cases, the outer surface of the MCs is relatively smooth. Some non-uniformity and wrinkles that might be observed are due to the fast formation of a mature, stiff shell through the reaction of the active H sites with the highly reactive isocyanate compounds, followed by shrinking of the core [17]. Another reason might be the inhomogeneous reaction kinetics and fluid-induced shear forces [5,11]. The inner surface is generally smoother since little shear flow occurs inside the MCs during the formation of the shell wall.

**Figure 5:**
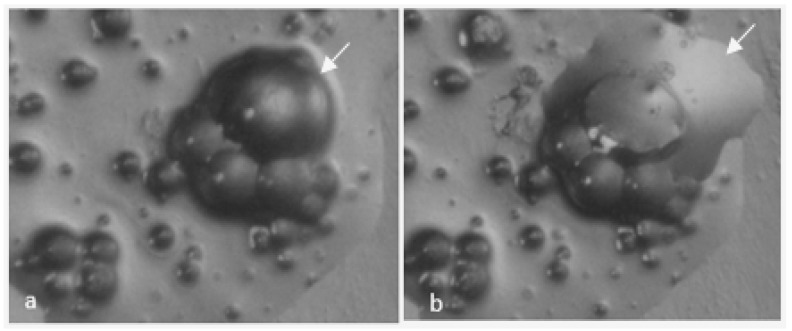
Optical microscopy images: (**a**) I MCs before tearing of the shell by the point of a needle; and (**b**) the same image after breaking the shell, exhibiting release of the core content (IPDI).

By optical microscopy, it was possible to follow a MCs’ shell formation behavior and to conclude about the shortest reaction time, for each synthesis, which allows enough maturity of the shell material to withstand the filtration step without damage of the MCs. In Figure 5, it is possible to observe that the MCs’ shell broke due to the pressure applied and a significant amount of liquid isocyanate (IPDI) was released from the MCs, confirming the success of the encapsulation process. It should be stressed that there is no solvent inside the capsules.

**Figure 6:**
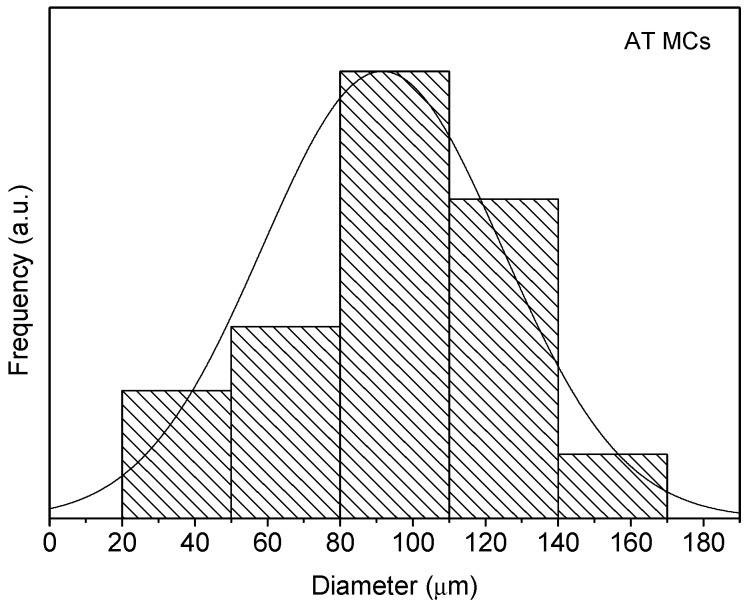
Size distribution of AT MCs.

**Figure 7:**
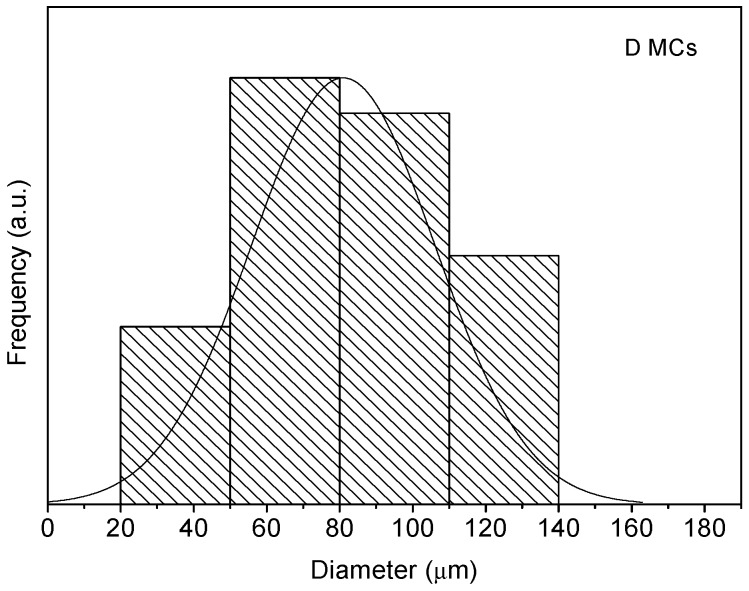
Size distribution of D MCs.

**Figure 8:**
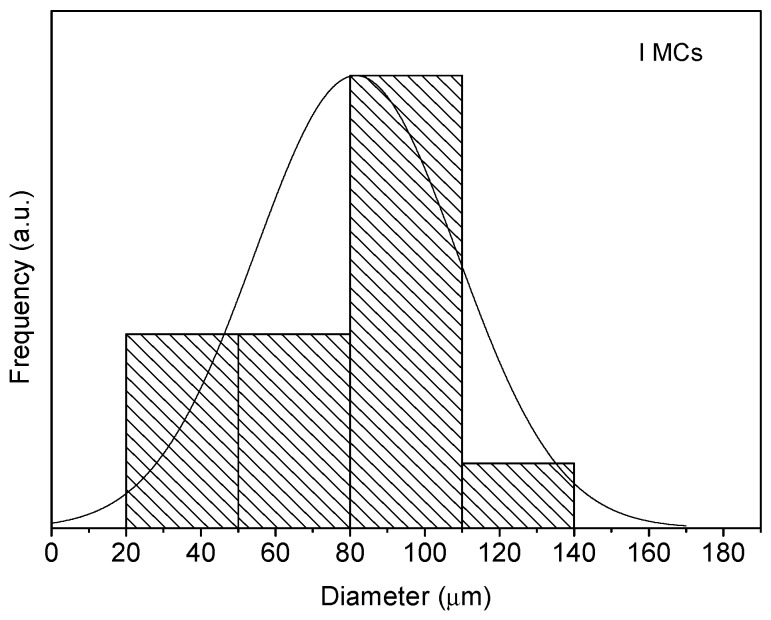
Size distribution of I MCs.

Through the analysis of the size distribution of the MCs, using the ImageJ software, it was possible to conclude that the particles have an average diameter of 95 ± 7, 81 ± 4 and 82 ± 7 μm for the AT MCs, D MCs and I MCs samples, respectively. For the stirring rate employed (400 rpm), the size of the MCs is herein found to be smaller than that reported in the literature, for instance by Yang et al. [5]. Indeed, besides shear/stirring rate, it is well known that the size of MCs is influenced by several factors including the viscosity of the dispersed and dispersant phase, interfacial tension of the media, the geometry of the mixing device, blade hydrodynamics, temperature, and surfactant effects [5,11,18,19]. In all cases, it is possible to state that the particle size distribution closely follows a normal distribution, because all the distributions had the characteristic bell-shaped curve, as observed in Figure 6, Figure 7 and Figure 8, and the *p*-values obtained through the Shapiro–Wilk normality test, listed in Table 2, were all higher than the defined alpha value of 0.05, not rejecting the null hypothesis that the values are normally distributed.

**Figure 9:**
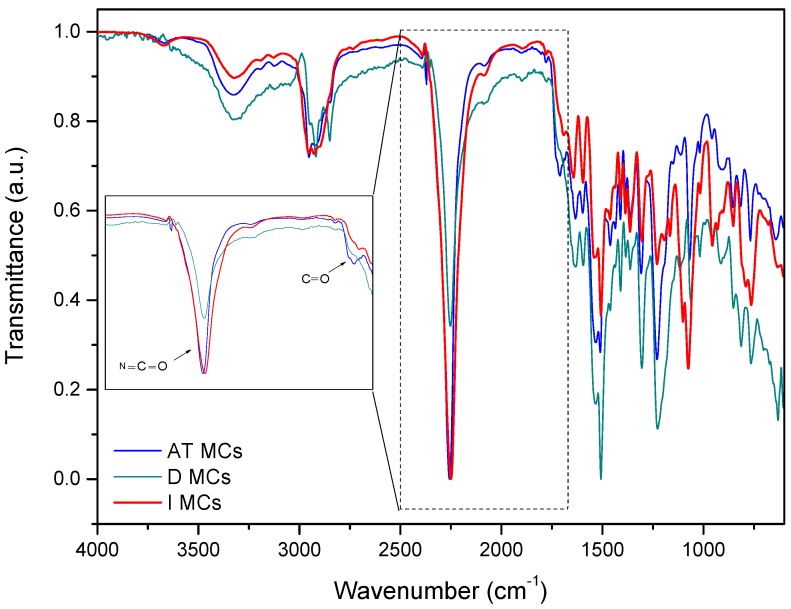
FTIR spectra of MCs obtained in this work. The inset shows a magnification of the peaks assigned to isocyanate NCO bond stretching and to the carbonyl (C=O) group present in urea and urethane moieties.

The FTIR spectra of IPDI and the IPDI loaded MCs were obtained to further reveal both the composition and the IPDI encapsulation effectiveness. From the FTIR spectra, shown in Figure 9, it is possible to observe an intense band peaked at ca. 2260 cm^−1^, related to the N=C=O bond stretching vibration, which indicates the presence of unreacted NCO groups in the MCs, confirming a successful encapsulation of IPDI. The peaks ascribed to the presence of amine groups can be observed at 3200–3400 cm^−1^ from N–H stretching of the amine bonds and at 1510 cm^−1^ from N–H bending. The presence of amine groups in the MCs can be related with the presence of PUa in the shell composition, derived mainly from the reaction of NCO groups with the active H sources used in the synthesis of AT MCs and D MCs, namely aminosilane and DETA, respectively. The carbonyl groups observed at ca. 1715–1730 cm^−1^ (C=O from urethane) and at 1680–1700 cm^−1^ (C=O from urea), the C=C groups detected at 1522 cm^−1^, the C–O–C group at 1214 cm^−1^ and C-O stretching at ca. 1300 cm^−1^ confirm the presence of urethane and urea linkages, evidencing that the MCs’ shell is composed by PU and PUa. Moreover, the peak assigned to the carbonyl group of urea is more intense than the same peak related to urethane bonds, which means that the majority of the shell material is indeed polyurea.

The peak at 1070 cm^−1^, typical of Si–O–Si asymmetric stretching vibrations, confirms the presence of siloxane Si–O–Si moieties in the shell, derived from the silanes employed in the synthesis, in the cases of AT MCs and I MCs. During the synthesis of AT MCs, the aminosilane and TEOS, when incorporated into the aqueous phase, tend to hydrolyze, forming silanol groups which, by polycondensation reactions form siloxane moieties (FTIR peak assignment at ca. 1070 cm^−1^), and when located at the droplets’ surface, can also react with NCO groups generating urethane moieties. Moreover, the NH groups from the aminosilane tend to react with the NCO groups at the interface of the aqueous/organic phases, originating urea moieties. The presence of silanes, as active H source, is found to result in a PUa/PU–silica hybrid shell material. The siloxane moieties are known to contribute to an improved mechanical strength and thermal resistance of the resulting shell.

Regarding the synthesis of D MCs, at which DETA was added as an active H source, the success of the IPDI´s encapsulation can be explained by the reaction of the amine groups of DETA with –NCO groups of the Ongronat^®^ 2500, leading to a fast formation of a PUa shell. 

For the I MCs synthesis, IPES was added to the organic phase. IPES contains a double organic functionality, namely –NCO and ethoxy (OCH_2_CH_3_) groups, and can be used as a crosslinker, contributing to the maturity and cohesiveness of the shell. In fact, once the NCO groups from IPES react with the OH groups from the aqueous phase, this molecule will be kept at the interface of the organic/aqueous phases, where the IPES´s alkoxide groups (OCH_2_CH_3_) suffer hydrolysis, forming silanol groups, which will react with the NCO groups from Ongronat^®^ 2500. The fact makes IPES to be considered a “latent” active H source, which also displays NCO functionality. In this sense, one can state that IPES acts as an active H source, which can be added to the organic (isocyanate containing) phase, contrary to most of the active H sources, establishing links with either the organic or the aqueous phases, due to its double organic functionality. The presence of IPES inside the tiny droplets of the emulsion, at a concentration of 25 wt.% in the O phase, results in a significant concentration at the interface of the organic/aqueous phases, rather than if it had been added to the aqueous phase, which accounts for 93.3 wt.% of the total emulsion mass. The NCO groups from Ongronat^®^ 2500 also react with the aqueous phase, contributing to the formation of the shell material.

Further calculation was carried out to estimate a relative measure of the isocyanate encapsulation efficiency, by considering the peak at ca. 2260 cm^−1^ assigned to NCO stretching (area in the range of 1926 to 2444 cm^−1^) and the area of the peak at 1300 cm^−1^ assigned to C–O stretching, typically related to the PUa/PU shell material, which does not tend to suffer significant changes over time after synthesis of the MCs. The peaks area was calculated using OriginPro 9 software, and the relative encapsulation yields were calculated based on the equation below:

$$Y=\frac{Area_{\;2260\;cm^{-1}}}{Area_{\;1300\;cm^{-1}\;}}$$

where *Y* can be considered as a relative encapsulation yield, which represents an indirect measure of the encapsulation efficiency. $Area_{\;2260\;cm^{-1}}$ and $Area_{\;1300\;cm^{-1}}$ represent the areas of the peak related to the NCO group and the area of the peak related to C-O stretch from the shell material, respectively. The obtained *Y* values are 13.75, 5.84 and 18.48 for AT MCs, D MCs and I MCs, respectively, as reported in Table 3. The MCs synthesized using IPES as the active H source are the ones shown to have more IPDI encapsulated, which might be correlated with a fast reaction between the active H source and the NCO groups of Ongronat^®^ 2500, leading to a faster shell formation which creates a barrier between the NCO groups from IPDI and the surrounding water medium. Indeed, the incorporation of IPES, as a “latent” active H source, inside the tiny droplets of the microemulsion, is shown to be an effective strategy for the desired high encapsulation yield. Its NCO group is responsible for its concentration at the interface of the organic/aqueous phases, being therefore highly available to react through its double organic functionalities, establishing links with both the isocyanate phase and the aqueous phase. 

On the other hand, the remaining active H sources, employed in the synthesis of AT MCs and D MCs, were added to the dispersant (W) phase, which creates a “dilution” effect (since the W phase consists of 93.3 wt.% of the total emulsion), requiring some time for them to migrate to the emulsion droplet surface to react with the NCO groups. The aminosilane and TEOS have the advantage, in comparison to DETA, of having more affinity to the oil phase, before the hydrolysis of the alkoxy groups occur, leading to a faster migration to the oil droplets and consequent reaction with the NCO groups, despite the slower reaction kinetics of the silanol groups with the NCO groups in comparison with NH (from DETA). DETA, on the other hand, has an affinity to the aqueous phase and not so much tendency to move toward the isocyanate droplets surface.

**Figure 10:**
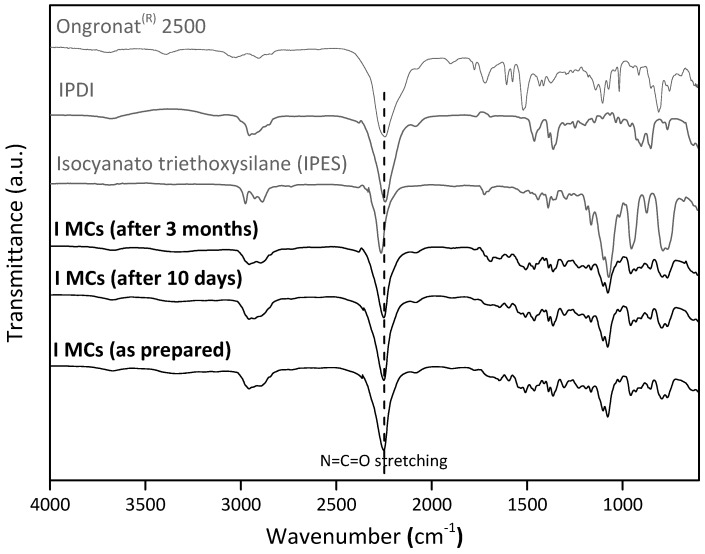
FTIR spectra of the I MCs as prepared, and 10 days and three months after their synthesis, to study the MCs shelf life. The spectra of IPES, IPDI and Ongronat^®^ 2500 are also represented for comparison.

Shelf-life studies were conducted on the I MCs (Figure 10) to confirm their moisture barrier capability. FTIR spectra of the MCs were taken at three different times after the MCs’ synthesis: right after the synthesis, and ten days and three months later. As it is possible to observe, the intensity of the NCO peak remains nearly constant over time, indicating a similar amount of liquid isocyanate inside the MCs and a good barrier effect by the shell.

The NCO peak shape and wavenumber, observed for the I MCs spectra, when compared to that of the isocyanate species employed in the synthesis, reveals that the encapsulated isocyanate is indeed IPDI, since the NCO peak of the Ongronat^®^ 2500 is much wider than the one observed in the I MCs spectra, and the NCO peak from IPES presents a deviation to larger wavenumbers.

TGA studies enabled the quantification of the liquid isocyanate in the MCs’ samples. Generally, water and other solvents evaporation occur during a first step, below 100 °C. The second degradation step is ascribed to the decomposition of encapsulated monomeric isocyanate, followed by a third and last step related to the polymeric, or hybrid, shell decomposition. It should be stressed that there is an overlap, resulting from the decomposition of isocyanate compounds (monomers, oligomers, and pre-polymer species), PU and PUa shell material, so there is not an exact borderline to separate them. There are references which mention that the PU decomposition starts around 200 °C [20], however, it was observed that the decomposition of isocyanate also occurs around this temperature [5,9,21]. In addition, it should be noted that the decomposition temperature range for the encapsulated isocyanate compounds is slightly different from that of non-encapsulated ones, and such difference will depend on the shell thickness, composition, and structure [20,22]. 

**Figure 11:**
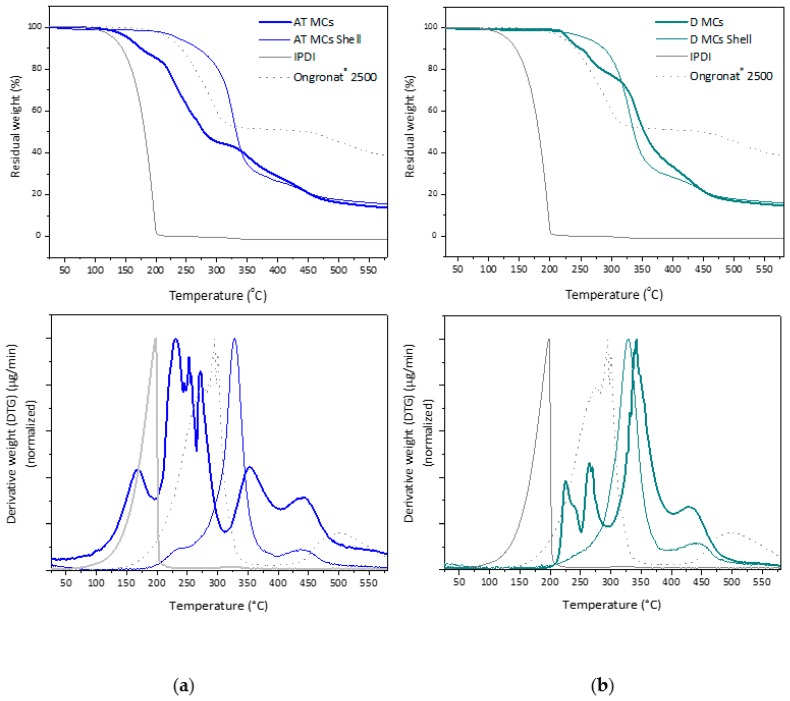
(**a**) Thermograms of the AT MCs, the respective shell and the isocyanates used in the synthesis (**top**) and respective first derivative curves (**bottom**); and (**b**) thermograms of the D MCs, the respective shell and the isocyanates used in the synthesis (**top**) and respective first derivative curves (**bottom**).

**Figure 12:**
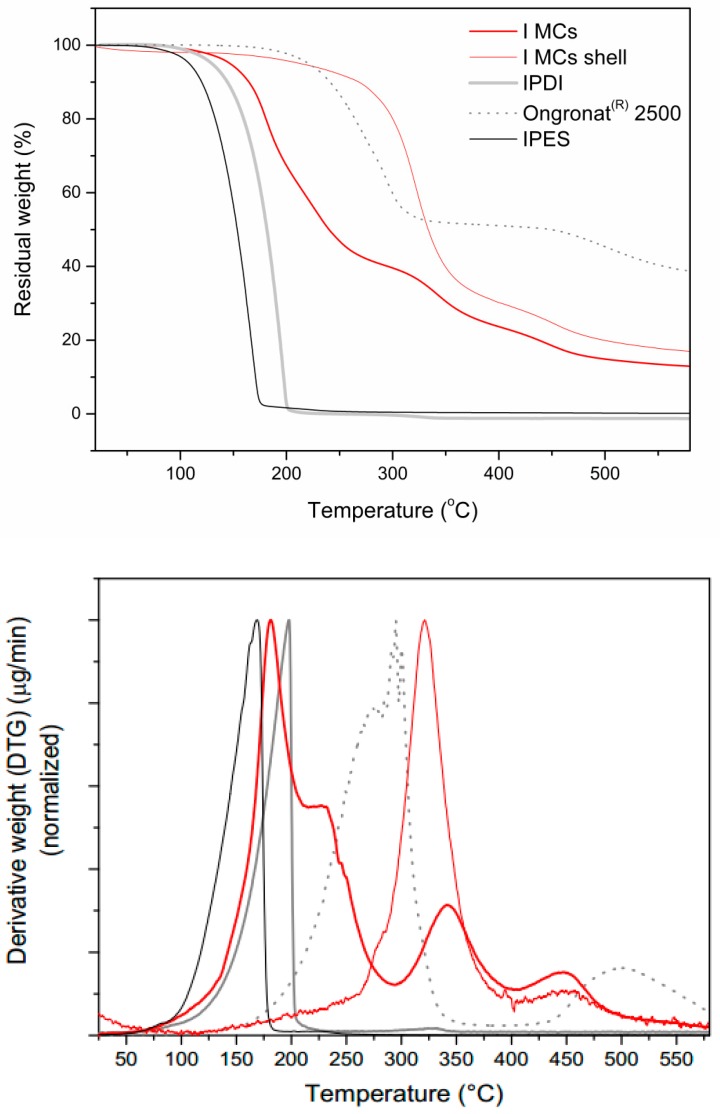
Thermogram of the I MCs, the respective shell and the isocyanates used in the synthesis (**top**) and respective first derivative curves (**bottom**).

Figure 11 and Figure 12 show the thermograms of the MCs, of the isocyanate species used in the syntheses and of the respective shells, as well as the corresponding first derivative curves. Indeed, it can be observed that, in the MCs’ shell thermograms, the PUa/PU decomposition tends to initiate at around 200 °C, as referred in the literature; however, it is only at 300 °C that a major mass loss is observed. The step of weight loss, in the range of ca. 150–300 °C, only present in the MCs thermograms, can be referred to the decomposition of encapsulated isocyanate compounds, but some care in the interpretation of the results should be taken because there starts to be an overlap with the shell material decomposition for temperatures above 200 °C, as observed in the first derivative curves. Contrary to some of the references found in the literature [8], the organic phase in the present study has no solvent at all, so that one can state that the mass loss observed above ca. 150 °C and below 300 °C is mainly due to pure encapsulated isocyanate. In fact, the decomposition temperature for pure IPDI is found to start around 100 °C, and it has been reported to occur in the range of 120–250 °C [5], which is found to happen in the thermograms of Figure 11 and Figure 12.

It should be noted that the thermograms exhibit different slopes between 150 and 300 °C, indicating that possibly there are isocyanates in different stages of polymerization, i.e., monomeric (IPDI and MDI) species and oligomeric (pre-polymeric) species. 

Regarding the shell, its decomposition starts around 300 °C and two steps can be observed in the thermograms: a first step (300–370 °C) related to the decomposition of soft segments and a second step (above 370 °C) related to the decomposition of hard polymeric segments from PU and PUa parts of the shell [22]. These steps correspond to first derivative curves peaked at ca. 345 and 440 °C. Considering these findings, it was possible to calculate the amount of encapsulated isocyanate in the AT MCs, D MCs and I MCs, which correspond to 52.8%, 13.95%, and 63.3%, respectively, as reported in Table 3.

As shown in Table 3, the trend obtained through the FTIR spectra analysis is corroborated by the TGA results, regarding the amount of encapsulated isocyanate, being I MCs the ones with higher IPDI encapsulation.

**Figure 13:**
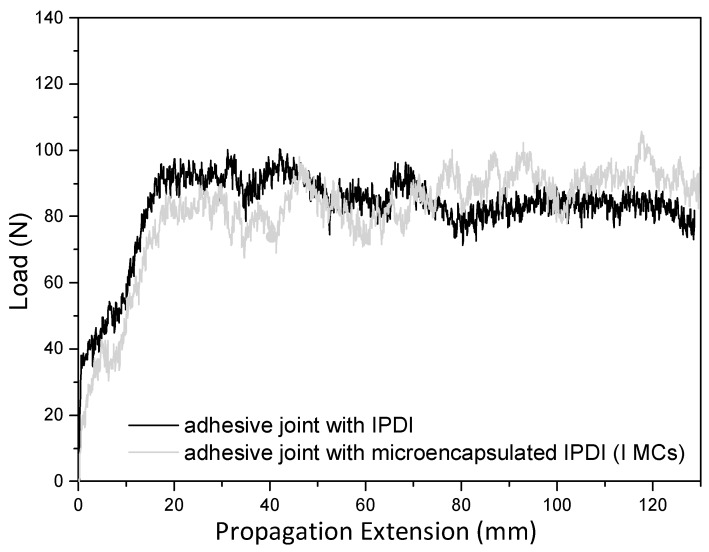
Peeling strength tests result. Load versus propagation extension for adhesive joints with IPDI and microencapsulated IPDI (I MCs), as crosslinkers.

As discussed before, the peeling strength test was performed as an indirect way to evaluate the behavior of the MCs in terms of isocyanate release, crosslinking effectiveness and resulting adhesion capacity. The adhesive formulation base component (OH pre-polymer), when tested without any isocyanate compound, exhibits an adhesion performance by itself, but it leads to an adhesive type of failure when performing the peeling test. It should be noted that an effective crosslinking, by the reaction with NCO groups, is expected to lead to a structural type of failure of the adhesive joint, which is the one that denotes a stronger adhesion performance. This is critical for the high adhesive performance and durability, desired in the footwear industry. The average load measured per unit width of the bond, at the peeling strength tests, is listed in Table 4, together with the type of failure observed during the tests. The same quantity of isocyanate, either encapsulated (in the form of I MCs) or non-encapsulated, was added to the OH pre-polymer, and the resulting adhesive joints were tested and compared with the OH pre-polymer (base component) without any isocyanate (crosslinker). It is shown, in Figure 13, that both adhesive joints having IPDI, either encapsulated or non-encapsulated, exhibit the same peeling strength, i.e., an average load per unit width of bond of ca. 3 N/mm, which denotes that the MCs are releasing the IPDI under the thermal and pressure stimuli, employed during the sample preparation, following the procedure used in the industry. According to internal information from CIPADE S.A., an adhesive, to be allowed for commercialization, must exhibit a peeling strength value equal or superior to 3 N/mm. Moreover, a structural and cohesive failure of the adhesive joint was observed for the sample with I MCs, which is an indicator of these MCs potential for the current application, i.e., for the next generation of mono-component adhesives.

## 4. Conclusions

Perfectly spherical, core–shell and free-flowing MCs, of PUa/PU polymeric shell or PUa/PU-silica hybrid shell, with average diameters ranging from 81 ± 4 to 95 ± 7 μm, were synthesized using an O/W emulsion combined with interfacial polymerization and exhibit a high loading of pure IPDI in the core. It was concluded that an enhanced encapsulation of pure IPDI isocyanate (above 60 wt.% of the MCs’ weight) occurs when the following strategies are used together: (a) mixture of IPDI with a small amount of commercially available Ongronat^®^ 2500, an isocyanate source that consists of monomeric and polymeric MDI species, and exhibits a significantly higher reactivity than IPDI, contributing for the fast shell formation; and (b) incorporation of a “latent” H active source, isocyanato triethoxysilane (IPES), in the organic phase (tiny droplets of the emulsion), which tends to be mainly concentrated at the interface of the organic/aqueous phases, and establishes links either with the aqueous phase (through its NCO groups) or with the organic phase (through its Si-OH groups). These PUa/PU-silica hybrid shell MCs exhibited approximately the same isocyanate content three months after their synthesis, demonstrating an effective barrier capacity of the shell. Their performance as crosslinkers in adhesive formulations was tested through peeling strength analyses and compared with adhesive joints having the same amount of non-encapsulated IPDI. Samples containing the I MCs were found to exhibit the same peeling strength as the samples with non-encapsulated IPDI, which reveals the release and crosslinking ability of the encapsulated IPDI. Additionally, the I MCs were responsible for a structural failure of the adhesive joint, which illustrates the potential of these MCs as crosslinkers in mono-component, self-reactive, safer and eco-innovative adhesives for highly demanding applications.

## Figures and Tables

**Table 1:** MCs’ synthesis parameters: active H source employed, maximum reactional temperature and synthesis.

MCs Acronym	Water (W) Phase	Oil (O) Phase	Active H Source (wt.% of Total Emulsion)	Maximum Temp. (°C)	Duration (Hours)
AT	Water + GA	Ongronat^®^ 2500 + IPDI	Aminosilane (5.7 wt.%) + TEOS (1.5 wt.%) (added to the W phase)	65	4
D			DETA (5.0 wt.%) (added to the W phase)	70	2
I			IPES (1.7 wt.%) (added to the O phase)	60	3

**Table 2:** *p*-values for AT MCs, D MCs and I MCs analysis, obtained through the Shapiro–Wilk normality test.

MCs	Shapiro–Wilk *p*-Value
AT	0.302
D	0.336
I	0.578

**Table 3:** Relative encapsulation yield (*Y*) and mass loss (%) of encapsulated IPDI for each synthesis.

MCs	Relative Encapsulation Yield (*Y*) from FTIR	Mass Loss (%) from TGA (Amount of Pure Encapsulated Isocyanate)
AT	13.75	52.80
D	5.84	13.95
I	18.48	63.30

**Table 4:** Peeling strength tests’ results.

Crosslinker Added to the OH Pre-Polymer	Average Load Per Unit Width of the Bond	Type of Failure Observed in the Peeling Strength Test
none	<2 N/mm	Adhesive, at the substrate/adhesive interface
IPDI	2.97 N/mm	Cohesive, through the adhesive
Microencapsulated IPDI (I MCs)	2.99 N/mm	Structural and cohesive rupture

## References

[1] Langenberg K.V., Warden P., Adam C., Milner H.R. (2010). The Durability of Isocyanate-Based Adhesives under Service in Australian Conditions. The Results from a 3 Year Exposure Study Accelerated Testing Regime (Literature Review).

[2] Hu M., Guo J., Yu Y., Cao L., Xu Y. (2017). Research Advances of Microencapsulation and Its Prospects in the Petroleum Industry. Materials.

[3] Duan B., Karsa D.R., Stephenson R.A. (2016). Microencapsulation via In Situ Polymerization. Handbook of Encapsulation and Controlled Release.

[4] Poland I., Datta S.S., Weitz D.A. (2014). Controlling the morphology of polyurea microcapsules using microfluidics. Langmuir.

[5] Yang J., Keller M.W., Moore J.S., White S.R., Sottos N.R. (2008). Microencapsulation of Isocyanates for Self-Healing Polymers. Macromolecules.

[6] Nguyen L.T.T., Hillewaere X.K., Teixeira R.F.A., Berg O.V.D., Prez F.E.D. (2014). Efficient microencapsulation of a liquid isocyanate with in situ shell functionalization. Polym. Chem..

[7] Credico B.D., Turri S. (2013). An efficient method for the output of new self-repairing materials through a reactive isocyanate encapsulation. Eur. Polym. J..

[8] Ming Y., Hu J., Xing J., Wu M., Qu J. (2016). Preparation of polyurea/melamine formaldehyde double-layered self-healing microcapsules and investigation on core fraction. J. Microencapsul..

[9] Li J., Mazumder M.A.J., Stover H.D.H., Sherly I.M., Hitchcock A.P. (2011). Polyurea microcapsules: Surface modification and capsule size control. J. Polym. Sci. A Polym. Chem..

[10] Sondari D., Septevani A.A., Randy A., Triwulandari E. (2010). Polyurethane microcapsule with glycerol as the polyol component for encapsulated self-healing agent. IJET.

[11] Kardar P. (2015). Preparation of polyurethane microcapsules with different polyols component for encapsulation of isophorone diisocyanate healing agent. Prog. Org. Coat..

[12] Loureiro M.V., Vale M., Schrijver A.D., Bordado J.C., Silva E., Marques A.C. (2018). Hybrid custom-tailored sol-gel derived microscaffolds for biocides immobilization. Microporous Mesoporous Mater..

[13] Loureiro M.V., Lourenço M.J., Schrijver A.D., Santos L.F., Bordado J.C., Marques A.C. (2017). Amino-silica microcapsules as effective curing agents for polyurethane foams. J. Mater. Sci..

[14] Loureiro M.V., Ciriminna R., Lourenço M.J., Santos L.F., Schrijver A.D., Bordado J.C., Pagliaro M., Marques A.C. (2017). Organically-modified silica based microspheres for self-curing polyurethane one component foams. Microporous Mesoporous Mater..

[15] Ciriminna R., Marques A.C., Bordado J.C., Schrijver A., Pagliaro M. (2014). Green-Caps: Towards solid curing agents for sustainable polyurethane foams. Sustain. Chem. Process..

[16] Almeida R.M., Marques A.C., Klein L., Aparicio M., Jitianu A. (2016). Characterization of Sol–Gel Materials by Infrared Spectroscopy. Handbook of Sol-Gel Science and Technology.

[17] Hano N., Takafuji M., Ihara H. (2017). One-pot preparation of polymer microspheres having wrinkled hard surfaces through self-assembly of silica nanoparticles. Chem. Commun..

[18] Huang M., Yang J. (2011). Facile microencapsulation of HDI for self-healing anticorrosion coatings. J. Mater. Chem..

[19] Tatiya P.D., Mahulikar P.P., Gite V.V. (2016). Designing of polyamidoamine-based polyurea microcapsules containing tung oil for anticorrosive coating applications. JCT Res..

[20] Brochu A.B., Chyan W.J., Reichert W.M. (2012). Microencapsulation of 2-octylcyanoacrylate tissue adhesive for self-healing acrylic bone cement. J. Biomed. Mater. Res. B Appl. Biomater..

[21] Yi H., Yang Y., Gu X., Huang J., Huan C.W. (2015). Multilayer composite microcapsule synthesized by Pickering emulsion templates and its application in self-healing coating. J. Mater. Chem. A.

[22] Koh E., Kim N.K., Shin J., Kim Y.W. (2014). Polyurethane Microcapsules for Self-Healing Paint Coatings. RSC Adv..

